# Supplementary material for: The Impact of Protein and Amino Acid Supplementation on Muscular Strength and Endurance in Recreational Gym-Goers During 8-Week Resistance Training
Source: Sports (Basel). 2025 Jun 11;13(6):182. doi: 10.3390/sports13060182 (PMC12197060; doi:10.3390/sports13060182)
Supplement: Supplementary file 1 [file sports-13-00182-s001.zip › sports-3625104-supplementary.pdf]

Table S1. Types of nutritional supplements and specific details regarding their consumption. CFM = cross-flow microfiltration, a cold-processing tool, chemical-free, that utilizes ceramic filters to remove lactose, fat, and other undesired whey constituents to isolate protein more effectively.

NS Type	Trade name	Formulation	Content	Administration
Whey Protein	Isolate Whey 100 CFM (Weider Sport and Specialty Nutrition, Iasi, Romania)	Powder<br>1 scoop with 30 g powder (with 25 g protein isolate) is dissolved in 200 mL of water	-25 g whey protein isolate<br>- Rich in BCAA (brain-chain-amino-acids) content<br>- 50 mg combined digestive enzymes (amylase, protease, cellulase, lipase, and lactase)<br>- Low-fat<br>https://www.weider.ro/magazin-proteine/proteine/isolate-whey-100-cfm-420gr-	For women:<br>1 sachet after training, recommended by the producer<br><br>For men:<br>2 sachets/day<br>1 sachet in the morning and<br>1 sachet<br>after training
Creatine	Premium Pure Creatine (Weider Sport and Specialty Nutrition, Iasi, Romania)	Powder<br>1 scoop contains<br>3.4 g creatine monohydrate (with 3000 mg creatine) is dissolved in 300 mL of water	- 3,4 g of creatine monohydrate per serving<br>(1 scoop) contains 3000 mg of creatine<br>- vegan<br>https://www.weider.ro/magazin-proteine/proteine/premium-creatine?gad_source	Recommended daily dose: 3000 mg creatine,<br>30 minutes before training
L-Carnitine	Carnitine 3000 (Martinez Nieto S.A., Cartagena, Spain) Obtained by Liposomal technology	Liquid<br>1 vial of 25 mL contains 3000 mg L-carnitine tartrate	- 3000 mg L-carnitine tartrate provides 2000 mg L-carnitine<br>https://www.marnys.ro/produse/l-carnitina-lipozomala-fiole-cu-3000-mg/	Recommended daily dose: 1 vial daily,<br>30 minutes before training
